# Seasonal variation in thermal tolerance of redside dace *Clinostomus elongatus*

**DOI:** 10.1093/conphys/coaa081

**Published:** 2020-08-28

**Authors:** Alexandra T A Leclair, D Andrew R Drake, Thomas C Pratt, Nicholas E Mandrak

**Affiliations:** 1 Department of Biological Sciences, University of Toronto Scarborough, 1265 Military Trail, Scarborough, Ontario M1C 1A4, Canada; 2 Department of Ecology and Evolutionary Biology, University of Toronto, 25 Wilcox Street, Toronto, Ontario M5S3B2, Canada; 3 Fisheries and Oceans Canada, Great Lakes Laboratory for Fisheries and Aquatic Sciences, 867 Lakeshore Road, Burlington, Ontario L7S 1A1, Canada; 4 Fisheries and Oceans Canada, Great Lakes Laboratory for Fisheries and Aquatic Sciences, 1219 Queen Street East, Sault Ste. Marie, Ontario P6A 2E5, Canada

**Keywords:** Climate change, endangered, long-term acclimation, phenotypic plasticity, short-term acclimation

## Abstract

Organisms living in environments with oscillating temperatures may rely on plastic traits to sustain thermal tolerance during high temperature periods. Phenotypic plasticity in critical thermal maximum (CT_max_) is a powerful thermoregulative strategy that enables organisms to adjust CT_max_ when ambient temperatures do not match thermal preference. Given that global temperatures are increasing at an unprecedented rate, identifying factors that affect the plastic response in CT_max_ can help predict how organisms are likely to respond to changes in their thermal landscape. Using an experimental thermal chamber in the field, we investigated the effect of short-term acclimation on the CT_max_ and thermal safety margin (TSM) of wild-caught redside dace, *Clinostomus elongatus*, (*n* = 197) in a northern population in Two Tree River, Ontario. Streamside CT_max_ trials were used to identify the maximum temperature at which redside dace maintain equilibrium, providing a powerful tool for understanding how thermal stress affects individual performance. CT_max_ and TSM of redside dace were sensitive to changes in temperature, regardless of season, suggesting that temperature pulses caused by climate change or urban activities can impose negative fitness consequences year round. Interestingly, an individual’s recent thermal history was more influential to its thermal tolerance than the current ambient water temperature. While the CT_max_ of redside dace increased with body size, the effect of body size on TSM remains unclear based on our models. The results provide insight into the thermal performance of redside dace that, to date, has been difficult to assess due to the species’ rarity and lack of suitable streamside protocols.

## Teaser

Climate change is increasing water temperature variability, with consequences for small-bodied fishes with limited migratory ability. We assessed seasonal variation in critical thermal maximum and thermal safety margin of an imperilled fish, demonstrating that its physiological ability to manage warming temperatures depends on its recent thermal history.

## Introduction

Ambient temperature is the principal environmental parameter governing physiological processes in ectotherms (Hochachka and Somero, 2002). The ability of organisms to physiologically adapt to changing temperatures is important for understanding which species can manage the threats posed by a warming climate (Seebacher *et al*., 2015). Average annual air and water temperatures are changing world-wide and causing global shifts in species distributions (Magnuson *et al*., 1990; Jackson and Mandrak, 2002; Perry *et al*., 2005; Ficke *et al*., 2007; Comte and Olden, 2017; IPCC, 2018). Contributing to these shifts is the elevated stress organisms face when thermoregulative strategies cannot respond as quickly as local temperatures. The lag between the changing environment and an organism’s capacity to respond has caused local extirpation in thermally sensitive species (Chu *et al*., 2005; Comte *et al*., 2013; Lynch *et al*., 2016). To best predict how climate change will affect species distributions, it is necessary to understand the effect of thermal stress on organism thermoregulation.

**Figure 1:**
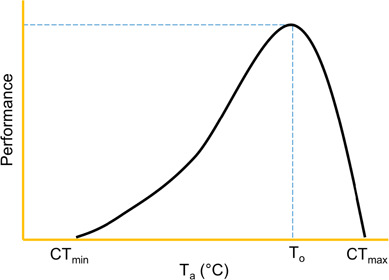
Theoretical thermal performance curve showing the final preferendum (T_o_), critical thermal minimum (CT_min_) and CT_max_, adapted from Huey and Kingsolver (1993).

Critical thermal maximum (CT_max_) is a quantitative measure of an organism’s thermal tolerance and represents the maximum temperature at which individual performance can occur (Schulte *et al*., 2011). CT_max_ can be several degrees higher than an organism’s preferred temperature and is commonly depicted on a thermal performance curve as a function of ambient temperature (T_a_) (Fig. 1; Huey and Kingsolver, 1993). As T_a_ approaches CT_max_, metabolic efficiency and fitness rapidly decline (Brown *et al*., 2004; Dowd *et al*., 2015; Molnar *et al*., 2017). Given this response, organisms living in environments with oscillating temperatures may rely on plastic traits to sustain thermal tolerance during high temperature periods (Gabriel *et al*., 2005; Gunderson and Stillman, 2015). Phenotypic plasticity in CT_max_ is a powerful thermoregulative strategy that enables organisms to adjust CT_max_ when ambient temperatures do not match thermal preference. Given that global temperatures are increasing at an unprecedented rate (IPCC, 2018), identifying factors that affect the plastic response in CT_max_ can help predict how organisms are likely to respond to changes in their thermal landscape.

CT_max_ can change based on environmental or biological factors. Environmental factors (e.g. ambient temperature, dissolved-oxygen) have a generally consistent effect among taxa, whereas the effects of certain biological factors (e.g. ontogeny, body size) can be species-specific. Increases to T_a_ will causally increase CT_max_, although not proportionally (Lutterschmidt and Hutchison, 1997; Ficke *et al*., 2007) and will eventually cause CT_max_ to asymptote at a temperature unique to each species (Kilgour and McCauley, 1986; Kita *et al*., 1996; Currie *et al*., 1998; Zhang and Kieffer, 2014; McDonnell and Chapman, 2015). This relationship is observed in most, but not all, species (Baroudy and Elliott, 1994). Similarly, increases to dissolved-oxygen concentration causally increase CT_max_ given its fundamental role in circulatory and respiratory function (Chretien and Chapman, 2016). Conversely, CT_max_ can both increase and decrease with life stage (Imsland *et al*., 1996; Elliott and Elliott, 2010; Komoroske *et al*., 2014) or body size (Recsetar *et al*., 2012; Zhang and Kieffer, 2014). This is contrary to the belief that small-bodied fish warm faster than large-bodied fish given the difference in tissue mass (Recsetar *et al*. 2012; Messmer *et al*. 2017). Therefore, changes in environmental factors, on average, have a more consistent effect on changes in CT_max_ than do biological factors. Despite the importance of environmental factors on CT_max_ plasticity, and the role of plasticity in CT_max_ in coping with increased thermal stress, relatively little research have investigated whether seasonal oscillations in T_a_ can invoke a plastic response during the year.

Given that temperature oscillates both daily and seasonally, it is likely that organisms having plasticity in CT_max_ will exhibit varying CT_max_ during the year. The difference between CT_max_ and T_a_ indicates how closely an organism lives to its thermal limit within the environment. This difference, known as the thermal safety margin (TSM), can be used to study how organisms will likely respond to temperature pulses throughout the year (Sunday *et al*., 2014; Shultz *et al*., 2016). As ambient temperatures increase, CT_max_ will approach its asymptote and the TSM will narrow, creating a period of heightened sensitivity to acute increases in temperature. Identifying when organisms experience a narrow TSM as a function of natural warming and cooling cycles can help predict the lethality of local temperature pulses or anomalies associated with climate change. Therefore, determining ecological factors that affect CT_max_ and TSM is necessary to identify periods in the growing season when organisms are most sensitive to acute temperature changes.

Laboratory experiments are conventionally used to measure CT_max_ and are an effective method to identify the extent of an organism’s CT_max_ plasticity (Lutterschmidt and Hutchison, 1997). However, the design of laboratory experiments typically does not account for the variation in temperature that organisms in the wild experience during seasonal warming and cooling and other natural temperature oscillations. The constant acclimation temperatures used in laboratory experiments may not reflect natural, short-term temperature changes and can over- or under-estimate the CT_max_ of organisms at discrete points in the year (Denny and Dowd, 2012; Ruel and Ayres, 1999; Vasseur *et al*., 2014). For this reason, CT_max_ obtained from laboratory experiments can be problematic when interpreting seasonal changes to the TSM or predict sensitive periods during the organism’s growing season. Alternatively, field CT_max_ experiments conducted *in situ* can capture the natural variation in ambient temperature and assess CT_max_ in the presence of other ecological factors (e.g. predators, food availability). Field experiments also reduce uncertainty about the range of temperatures that organisms experience each season and can allow for different lengths of acclimation to be evaluated. For these reasons, our study uses field-based CT_max_ experiments to assess how the TSM of a wild fish population can change in response to local warming events *in situ* as a function of CT_max_ plasticity.

Redside dace, *Clinostomus elongatus*, is a coolwater minnow native to eastern North America. The species is threatened by several anthropogenic activities (e.g. urbanization, non-native species, climate change; COSWEIC, 2017; DFO, 2019) throughout its Canadian range. Urbanization has been identified as the main factor for the rapid extirpation of redside dace in the core of its Canadian range in the Lake Ontario drainage within the Greater Toronto Area (Poos *et al*., 2012; COSEWIC, 2017; DFO, 2019). Its small body size and specific habitat requirements limit dispersal, which has expedited local extirpations in habitats facing harmful conditions (Poos and Jackson, 2012; Poos *et al*., 2012; Drake and Poesch, 2020). Given that habitat perturbations associated with urbanization or agriculture (e.g. urban and agricultural run-off, effluent discharge) can cause warm pulses in urban waterbodies (McGurk, 1989; Klapper, 1991; Van Buren *et al*., 2000; Thompson *et al*., 2008; Madden *et al*., 2013), determining how redside dace CT_max_ and TSM change throughout the year is crucial for predicting the negative effects of urban development on individual capacity to grow, disperse and reproduce. This information is also imperative for informing mitigation strategies and recovery actions, as it will identify periods when redside dace will be particularly vulnerable to temperature changes. Plasticity in CT_max_ of redside dace has only been studied for a subset of acclimation temperatures by Novinger and Coon (2000). While Novinger and Coon (2000) indicated that redside dace is capable of CT_max_ plasticity, the laboratory experiments did not examine how the species is likely to respond to acute temperature changes across seasons based on temperature variability encountered in the wild.

The objectives of our study are to assess how seasonal water temperature variation can affect the CT_max_ and TSM of redside dace *in situ*, specifically: (i) the effect of short- and long-term acclimation on CT_max_ and TSM and (ii) the role of body size on these responses. We hypothesize that: (i) redside dace exposed to naturally varying ecological conditions will demonstrate a different CT_max_ in each season, (ii) redside dace will display a narrower TSM as T_a_ increases and (iii) small-bodied redside dace will have a lower CT_max_ and narrower TSM than large-bodied redside dace based on the expectation that small-bodied fish warm faster than large-bodied fish given the difference in tissue mass (Becker and Genoway, 1979).

## Materials and methods

**Figure 2:**
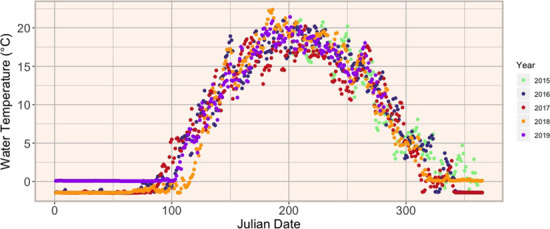
Mean daily ambient water temperature of Two Tree River, Ontario, June 2015–October 2019 (unpublished data from Derek Goertz, Ontario Ministry of Natural Resources and Forestry).

Our study analysed monthly changes in the CT_max_ and TSM of redside dace in Two Tree River, a northern Lake Huron tributary that bisects the rural residential St. Joseph’s Island and is the northernmost locality and least anthropogenically influenced population of redside dace in Canada (COSEWIC, 2017). Two Tree River has a streambed primarily comprised of clay and silt, riparian zone dominated by herbaceous plants and shrubs, a broad range of water temperatures (Fig. 2) and less human development than other redside dace habitats in southern Ontario. Therefore, Two Tree River is an ideal system to assess seasonal plasticity in CT_max_ in the absence of other threats (e.g. urbanization).

Data were collected 1 week per month June–October 2018 and May and October 2019 to measure CT_max_ and TSM from different open-water acclimation periods. This approach increased the likelihood of capturing acclimation states that may occur as a function of seasonal warming and cooling. Data were not collected from November 2018 to April 2019 because snow and ice limited stream access, and only May and October were sampled in 2019 to cover gaps in ambient water temperatures in the 2018 sampling. A Hobo Water Temperature Pro V2 data logger was placed in Two Tree River near the sample site to monitor hourly changes in ambient water temperature throughout the study period.

### Fish sampling

Redside dace (*n* = 197) was captured during daylight hours with a bag seine (9.14 m long, 0.63 cm mesh size) or backpack electrofisher (Halltech HT2000) within a 200 m reach of Two Tree River. Backpack electrofishing was the most effective sampling method in May and October due to the inability to seine effectively in high water levels. Collected fish were held in Two Tree River in a covered flow-through bin [8.3 L bin for juveniles (35–50 mm T_L_), 45 L bin for adults (51–127 mm T_L_)] near the experimental site and allowed a minimum recovery period of 30 minutes before beginning a CT_max_ trial. Holding times in the flow-through bin varied between months due to changes in trial length, but no fish was held longer than 50 hours. Any redside dace not tested on the date of capture remained in the flow-through bin until trials resumed the following morning. Given the holding bin ensured direct water flow with the surrounding waterbody, the water chemistry and temperature in the holding bin was assumed to be consistent with the river. No mortalities occurred inside the holding bin.

### Critical thermal maximum

CT_max_ trials were conducted on 30 fish per week between 0700 and 1900 hours for all months sampled, except in June 2018 (21 fish), September 2018 (28 fish) and October 2018 (29 fish). Sample sizes were reduced in these months because of either difficulties capturing fish (June and September 2018) or difficulty differentiating visual cues as fish neared CT_max_ (October 2018). The CT_max_ of redside dace was quantified using an approach similar to Chretien and Chapman (2016). All water for trials was collected directly from Two Tree River to ensure each fish maintained its natural thermal history immediately prior to the start of the trial. Prior to each trial, a 37.8 L glass aquarium (64.14 cm × 26.67 cm × 32.00 cm), with a mesh holding box (25.91 cm × 15.24 cm × 15.49 cm) fastened to the tank’s inner wall, was filled with river water. The aquarium was located on the stream bank adjacent to the collection site. The mesh holding box served as a protective barrier between the immersed portion of the JULABO CORIO CD Heating Immersion Circulator and the fish. An EXO3 Multi-Parameter Sonde monitored dissolved-oxygen concentrations during trial to ensure critical levels (<5 mg DO/L, Saari *et al*., 2018) were not exceeded.

Each trial began with the transfer of a single redside dace from the flow-through bin to the holding box. After a 5 minute acclimation period, the immersion circulator increased tank water temperature at a constant rate of 0.33 °C per minute until equilibrium was lost. Although this warming rate is unlikely to occur in the wild, it is commonly used in CT_max_ experiments (Becker and Genoway, 1979; Currie *et al*., 1998, 2004; McDonnell and Chapman, 2015; Chretien and Chapman, 2016; Di Santo and Lobel, 2017). Loss of equilibrium for 5 seconds was used as the trial endpoint, similar to other studies (McDonnell and Chapman, 2015). The 5-second duration provided an acceptable balance between the ability to reliably observe the trial endpoint while minimizing prolonged physical harm. After the trial, the redside dace was immediately removed from the tank, measured for total length using a Wildco measuring board (model # 118-E40, 0–360 mm), fin clipped and placed in a recovery bucket containing 50% tank water and 50% stream water to reduce thermal shock. Fin clips were taken as a precautionary measure to ensure individual redside dace were not retested. When fully recovered, the redside dace was returned to Two Tree River.

The experiment was conducted in accordance with animal care guidelines of the University of Toronto Animal Care Committee (AUP 20012425) and Fisheries and Oceans Canada (AUP 1852).

### Statistical analyses

To test the hypotheses that (i) redside dace exposed to natural ecological conditions will demonstrate a significantly different CT_max_ and TSM each season and (ii) TSM will narrow as T_a_ increases, we first checked for outliers, homogeneity, a normal distribution and collinearity (Zuur *et al*., 2010). We then used a pairwise Wilcoxon rank of sums test (non-parametric repeated measures analysis of variance) to assess monthly differences in T_a_, CT_max_ and TSM. TSM was calculated by subtracting the daily maximum water temperature of Two Tree River on date of trial (Thab_max_) from the respective trial’s CT_max_. Data transformation failed to normalize CT_max_, T_a_ and TSM; therefore, pairwise Wilcoxon rank of sums test was used.

**Figure 3:**
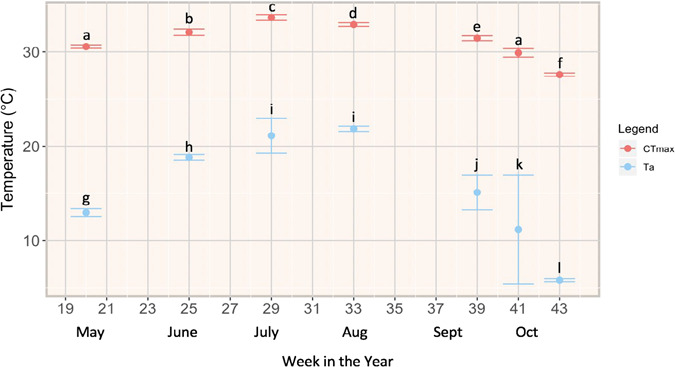
Mean acclimation temperature (T_a_, blue) and critical thermal maxima (CT_max_, red) of Redside Dace captured in Two Tree River in 2018 (June–October) and 2019 (May, October); data are presented by Julian week to demonstrate seasonal changes in CT_max_; standard errors (±SEM) were calculated from variance of monthly means (letters indicate significance between months as calculated by pairwise Wilcox tests).

To test the hypothesis that small redside dace will have a lower CT_max_ and narrower TSM than large redside dace, we used two sets of general linear mixed models (GLMMs) to identify the factors that predict CT_max_ and TSM. First, a short-term acclimation covariate was determined by calculating daily mean water temperature 1–14 days prior to trial and introducing the values as single covariates to a candidate GLMM model set for CT_max_ and TSM. The model of best fit in both model sets, determined by deviation information criterion (DIC) (Hadfield, 2010; Healy *et al*., 2014), identified the daily range of temperatures most relevant to the measured variation in CT_max_ and TSM. This daily temperature range defined our short-term acclimation covariate. A long-term acclimation covariate was defined as the mean temperature 12 weeks prior to trial to capture acclimation history from the previous season. A body size covariate was defined as the total length (mm) of each fish. These three covariates, including short-term acclimation, were then added to a second set of GLMMs to predict CT_max_ and TSM. Stepwise model selection removed parameters until a final parsimonious model was identified, as determined by DIC ranking. The random effects for all GLMMs were defined as Julian date, time of trial (morning, afternoon, evening) and time in holding bin (< or > 12 hours) to control for non-independence in CT_max_ and TSM associated with, respectively, the repeated sampling of the same population, diel changes in metabolism and changes in body condition as a function of time in the flow-through holding bin.

We fit models using Bayesian GLMMs from the MCMCglmm R package (Hadfield, 2010). The MCMCglmm package applies a Markov chain Monte Carlo estimation approach by using multiple simulations to determine the posterior distribution of CT_max_ and TSM (Hadfield, 2010). Each model ran 13 000 intervals with a thinning value of 10, a burn-in of 3000 and was verified that there was no autocorrelation. All analyses were performed in R v3.5.3 using the RStudio v1.1.3 interface (R Development Core Team, 2018; RStudio Team 2020).

## Results

CT_max_ of redside dace in Two Tree River differed significantly by month and increased disproportionally with T_a_ (Fig. 3). All means differed significantly from one another with exception of mean CT_max_ in May and October 2019 (*P* = 0.54) and mean T_a_ in July and August 2018 (*P* = 0.14). Mean CT_max_ ranged from 27.59 ± 0.16 C (October 2018) to 33.64 ± 0.31 C (July 2018) and mean T_a_ ranged from 5.79 ± 0.15 C (October 2018) to 21.85 ± 0.28 C (August 2018).

**Figure 4:**
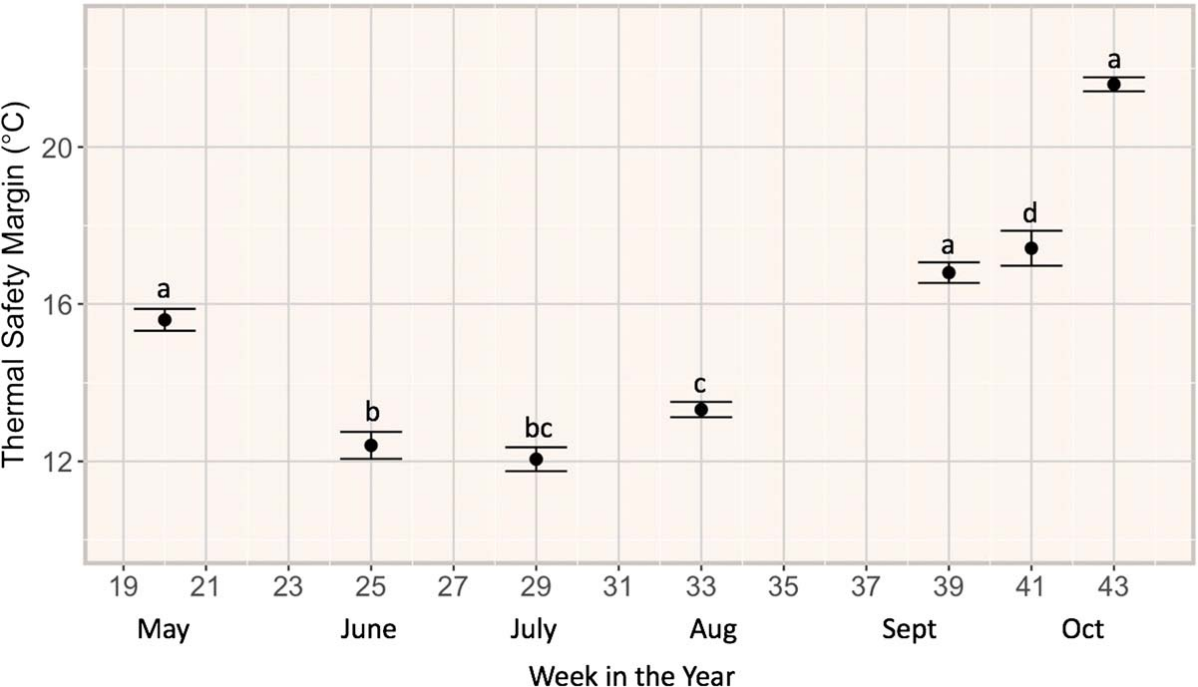
Mean thermal safety margin (TSM) of Redside Dace captured in Two Tree River in 2018 (June–October) and 2019 (May, October); data are presented by Julian week to demonstrate seasonal changes to TSM; standard errors (±SEM) were calculated from variance of monthly means (letters indicate significance between months as calculated by pairwise Wilcox tests).

Mean TSM was generally narrower in the summer, when T_a_ was high and CT_max_ was near its maximum, than in other seasons (Fig. 4). All means were significantly different with exception of mean TSM in May and September 2018 (*P* = 0.37), June and July 2018 (*P* = 0.13) and July and August 2018 (*P* = 0.78). Mean TSM ranged from 12.01 ± 0.34 C (June 2018) to 21.59 ± 0.18 C (October 2018).

Of the short-term acclimation candidate model set for CT_max_, the 2-day GLMM best predicted the observed variance as determined by the DIC ranking (718.9; Table 1). Of the short-term acclimation candidate model set for TSM, all mean daily temperatures were equally able to predict the observed variation with exception of 1-day mean T_a_ (Table 2). Given the clear significance of the 2-day mean T_a_ for the CT_max_ GLMM, and its inclusion in the TSM parsimonious models, this term was used to represent the short-term acclimation covariate in the second set of GLMMs. In both the CT_max_ and TSM GLMM candidate model sets, T_a_ was not significant. All models less parsimonious than T_a_ were excluded from Tables 1 and 2.

**Table 1:** Results of the GLMMs of CT_max_ based on different short-term acclimation periods for redside dace in Two Tree River

**Short-term acclimation**	**DIC**	**∆DIC**
2 days	718.9	0
3 days	724.2	5.24
4 days	726.5	7.53
7 days	726.6	7.65
12 days	726.9	7.86
14 days	727.4	8.43
11 days	727.9	8.86
5 days	728.4	9.41
8 days	729.7	10.67
6 days	730.8	11.85
10 days	731.8	12.83
13 days	732.2	13.17
9 days	733.8	14.82
T_a_	746.2	27.24

Temperature at start of trial (Ta) and mean temperature 2–14 days prior to trial (2–14 days) are listed according to DIC and ∆DIC.

**Table 2:** Results of GLMMs of TSM based on different short-term acclimation periods for redside dace in Two Tree River

**Short-term acclimation**	**DIC**	**∆DIC**
4 days	763.93	0
7 days	764.02	0.09
10 days	764.08	0.15
5 days	764.12	0.19
12 days	764.15	0.22
6 days	764.23	0.30
11 days	764.31	0.38
2 days	764.41	0.48
8 days	764.53	0.60
9 days	764.54	0.61
13 days	764.84	0.91
3 days	764.89	0.96
14 days	764.94	1.01
T_a_	782.45	17.52

Temperature at start of trial (T_a_) and mean water temperature 2–14 prior to trial (2–14 days) are listed according to DIC and ∆DIC.

Of the second candidate model sets that evaluated the effects of short- and long-term acclimation and body length, two parsimonious models for CT_max_ and numerous parsimonious models for TSM indicated that no one model could best predict the observed variation in either response variable (Table 3; ∆DIC < 2 units). These results support three possible conclusions: (i) all tested covariates have a significant effect on CT_max_ and TSM (CTM_2_, TSM_3_), (ii) long-term acclimation may not consistently have an effect on CT_max_ and TSM and (iii) body length may not consistently have a significant effect on TSM. However, in accordance with the principle of parsimony, we are inclined to accept CTM_1_ (which includes the 2-day mean T_a_ and body length) and TSM_1_ (which includes only the 2-day mean T_a_) as the final CT_max_ and TSM models, given our candidate model set.

**Table 3:** Results of CT_max_ and TSM GLMMs for 2-day short-term acclimation (2d), total length (L_t_; mm) and 12-week long-term acclimation (12w) for redside dace in Two Tree River

**Model**	**Model terms**	**DIC**	**∆DIC**
CTM_1_	2d + L_t_	715.99	0
CTM_2_	2d + L_t_ + 12w	717.82	1.83
CTM_3_	2d	718.93	2.94
CTM_4_	2d + 12w	720.79	4.8
CTM_5_	L_t_	726.40	10.41
CTM_6_	L_t_ + 12w	726.49	10.5
CTM_7_	12w	732.34	16.35
TSM_1_	2d	764.20	0.00
TSM_2_	2d + 12w	764.54	0.33
TSM_3_	2d + L_t_	765.23	1.03
TSM_4_	2d + L_t_ + 12w	765.32	1.12
TSM_5_	12w	766.33	2.13
TSM_6_	L_t_	767.47	3.26
TSM_7_	L_t_ + 12w	767.58	3.38

Models are listed by DIC and ∆DIC, ∆DIC < 2 units for significant models (CTM_1–2_ and TSM_1−5_).

## Discussion

We assessed whether seasonal water temperature variation in Two Tree River affected the CT_max_ and TSM of redside dace *in situ*. Our evaluation led to five key results. First**,** as predicted, the CT_max_ of redside dace differed significantly by month. This indicates that redside dace has a plastic response in its CT_max_ to cope with natural seasonal changes in water temperatures. Second, TSM of redside dace narrowed with increasing T_a_, which supports our second prediction and suggests redside dace experiences its upper thermal limit in the summer and, therefore, may be more vulnerable to high temperature fluctuations in summer than in other seasons. Third, a 2-day short-term acclimation period was the most significant variable explaining variation in CT_max_, indicating that the recent thermal history of redside dace can influence its current thermal tolerance. Fourth, short-term acclimation periods of 2–14 days are more significant to TSM than the current T_a_, indicating that recent thermal history is more influential to redside dace thermal tolerance than the current T_a_. Finally, multiple parsimonious models for the effect of short- and long-term acclimation and body size on CT_max_ and TSM limit our ability to identify a single optimal model. Regardless, the prediction that larger redside dace would have a higher CT_max_ was supported by the best fit model.

Because exposure to warm acclimation temperatures can improve thermal tolerance in many vertebrates (Beitinger *et al*., 2000; Chown and Treblanche, 2006), it was not surprising that CT_max_ increased with T_a_. As temperature increases, organisms may undergo molecular and cellular changes to maintain physiological rates, thereby matching their thermal tolerance to the environment (Clarke, 2003). However, the magnitude of increase in CT_max_ of redside dace did not match that of T_a_ in Two Tree River, indicating that seasonal changes to CT_max_ are restricted by biological processes. The metabolic theory of ecology indicates that all biological processes are dependent on organism metabolism, which relies on temperature-sensitive metabolic enzymes (Brown *et al*., 2004). Extreme temperatures can cause these enzymes to reversibly inactivate and halt metabolic processes (Molnar *et al*., 2017). Similarly, the oxygen- and capacity-limited thermal tolerance theory predicts that circulatory and respiratory activity will decline as a function of high metabolic demand in hypoxic environments (Chretien and Chapman, 2016). Therefore, a fish’s capacity to acclimate to increasing water temperatures may be restricted by their inability to acquire a sufficient amount of oxygen to maintain circulatory and respiratory activity. Both theories suggest that temperature-dependent biological processes can confine the responsive changes to CT_max_ and prevent thermal acclimation from occurring at the same rate as environmental temperature changes. In the current study, it remains unclear how the thermal response of redside dace may change at T_a_ beyond those measured. However, the disproportional increase in CT_max_ with T_a_ indicates that a point may be reached at which no further increase to CT_max_ is possible.

It was not surprising that TSM of redside dace was narrowest in the summer and broadest in the fall. From this, we conclude that redside dace lives near its upper thermal limit in the summer and may be more vulnerable to temperature pulses in June–August than other periods during the year. However, it is possible for redside dace to experience negative consequences to thermal fluctuations year-round depending on the magnitude of the thermal pulse. This challenges the common belief that temperature fluctuations are only of concern during the season with the highest temperatures, typically summer (Sandblom *et al*. 2016). Therefore, anthropogenic activities known to cause acute increases to water temperature (e.g. shoreline development, urban and agricultural run-off; McGurk, 1989; Klapper, 1991; Van Buren *et al*., 2000; Thompson *et al*., 2008; Madden *et al*., 2013) will have a lesser effect on redside dace if they can be mitigated and (or) restricted to cooler months of the year, when the difference between ambient water temperature and CT_max_ is largest.

The significant short-term acclimation periods were not consistent between our CT_max_ and TSM models, which may suggest recent thermal history can influence these aspects of the thermal response differently. The significant 2-day acclimation period for our CT_max_ model set suggests that the thermal response of redside dace is delayed by 48 hours to avoid a pre-emptive change to CT_max_. This may be an adaptive strategy to avoid responding to diurnal temperature oscillations or short-lived temperature anomalies. Our short-term acclimation model set for TSM indicated that no particular duration of thermal history 2–14 days prior to thermal stress was best able to explain this aspect of redside dace thermal response. However, given that our initial temperature and 1-day acclimation period models were not significant, we can conclude that recent thermal history does influence redside dace TSM, but the range of relevant past temperatures remains unclear. Interestingly, T_a_ was not an important predictor for both the CT_max_ and TSM models, indicating that the immediate thermal conditions are not as important in understanding redside dace thermal response as its recent (2–14 days prior) thermal history.

Given that our stepwise model selection produced several parsimonious models, there is uncertainty about how the tested covariates influence CT_max_ and TSM. CTM_1_ and CTM_2_ only differ by the inclusion of long-term acclimation. Therefore, we can confidently conclude short-term acclimation and body size have a significant effect on redside dace CT_max_. However, it is difficult to determine whether long-term acclimation has a significant effect on CT_max_. A similar issue was encountered with multiple competing TSM models, except that the significance of two covariates, body size and long-term acclimation, was unclear. Given that these covariates are not always present in our models, there is insufficient evidence to demonstrate their effect on TSM. Therefore, we accept TSM_1–5_ as being equally plausible models, but conclude that short-term acclimation is significant to both CT_max_ and TSM, given its inclusion in each parsimonious model.

Similar to Novinger and Coon (2000), our study indicates that redside dace demonstrates a plastic response in CT_max_. Given that redside dace dispersal can be limited by its strict habitat requirements and small body size (McKee and Parker, 1982; Novinger and Coon, 2000; Poos and Jackson, 2012; Drake and Poesch, 2020), phenotypic plasticity in thermal tolerance can serve as a powerful fitness tool that facilitates acclimation in thermally stressful conditions. Thermally stressful conditions are widespread and increasing in the Lake Ontario basin, primarily from urbanization, and causing the constriction of redside dace habitat in tributaries (COSEWIC, 2017). Run-off from impervious surfaces (e.g. roads, parking lots) and increased absorbance of solar radiation associated with land-use change (e.g. reduced riparian vegetation) can cause temperature pulses of 8.5 C or higher in urban waterbodies (McGurk, 1989; Thompson *et al*., 2008; Madden *et al*., 2013; Van Buren *et al*., 2000). If the change in temperature exceeds the TSM of redside dace, dispersal limitations (preference for slow-moving water, body size restrictions for long-distance movement, physical barriers) may lead to local extirpations (McKee and Parker, 1982; Nislow *et al*., 2011). Although the TSM range presented in this study (12.01 C–21.59 C) is higher than the noted temperature pulses, southern populations of redside dace may have a narrower TSM and lack the capacity to withstand temperature changes within its human-impacted habitat. For example, Turko *et al*. (in review) indicated that TSM in wild-caught redside dace in Ohio as 9.85 C–21.4 °C, indicating that narrower TSM may exist.

Considering the effect of water temperature on redside dace CT_max_ and TSM, mitigation strategies and recovery actions that maintain seasonally consistent water temperatures in known redside dace habitat will provide the greatest protection from thermal stress. This can be done in three ways. First, urban activities that can cause local temperature pulses (e.g. riparian removal) could be mitigated during summer when the TSM of redside dace is the narrowest by reducing impervious surfaces and changing farming practices (e.g. reduced tile drainage). This could avoid compounding thermal stress from shoreline construction with other year-round anthropogenic activities (e.g. stormwater drainage, agricultural run-off) to maintain more consistent seasonal thermal patterns in redside dace habitat. Second, riparian habitat removed along developed shorelines of known redside dace habitat could be restored to reduce the potential for abnormal water temperature fluctuations. Given our results, the CT_max_ of redside dace will likely plateau and, during high-temperature periods, riparian vegetation will provide necessary thermal refuge when redside dace is near its thermal limit. Third, establishing freshwater protection areas (FPAs) could help conserve and restore habitat and limit anthropogenic influences within highly urbanized communities (Saunders *et al*., 2002). FPAs would be most effective for populations residing in the Greater Toronto Area where development has significantly reduced available suitable habitat (COSWEIC, 2017; DFO, 2019). These actions may help maintain the TSM of redside dace, increase available thermal habitat and reduce the likelihood of local extirpations in urban waterbodies.

This study is the first to demonstrate the effect of variation in seasonal temperature on the thermal tolerance of wild redside dace *in situ*. We identified how thermal limits can change at different points in the growing season and demonstrated that redside dace is most sensitive to temperature variability in the summer, but can be affected by increasing temperatures throughout the year. Determining how thermal tolerance changes in response to seasonal and daily temperature cycles, and the conditions that increase the frequency of short-term warming, is necessary to predict the lethality of local temperature pulses or anomalies associated with climate change and other human-induced impacts such as urbanization. Our results indicate that redside dace in Two Tree River is not currently living at the edge of its thermal limit, but this does not negate the possibility that temperature pulses in urban waterbodies may threaten to surpass its TSM. Therefore, mitigation strategies and action plans that focus on maintaining seasonally consistent water temperatures in known redside dace habitat are expected to provide maximum protection against short-term thermal pulses.

## Funding

This research was funded by the Canadian Freshwater Species at Risk Research Network, a research partnership between Fisheries and Oceans Canada and participating academic institutions, including the University of Toronto Scarborough. Funding was also provided by Fisheries and Oceans Canada’s Species at Risk Program and Natural Sciences and Engineering Research Council of Canada Discovery Grant (grant number RGPIN-2014-05226).
